# Semaglutide in Atrial Arrhythmias: State of the Art and Future Perspectives

**DOI:** 10.3390/jpm16100506

**Published:** 2026-09-29

**Authors:** Enrico Maggio, Sara Trivigno, Marco Valerio Mariani, Agostino Piro, Nicola Pierucci, Antonio Vernile, Sara Vinciullo, Marta Palombi, Carmine Dario Vizza, Carlo Lavalle

**Affiliations:** Department of Medical and Cardiovascular Sciences, Sapienza University of Rome, Viale del Policlinico 155, 00159 Rome, Italy; enrico.maggio@uniroma1.it (E.M.); sara.trivigno@uniroma1.it (S.T.); marcoval.mariani@gmail.com (M.V.M.); a.piro@policlinicoumberto1.it (A.P.); nicola.pierucci@uniroma1.it (N.P.); antonio.vernile@uniroma1.it (A.V.); sara.vinciullo@uniroma1.it (S.V.); dario.vizza@uniroma1.it (C.D.V.)

**Keywords:** semaglutide, atrial fibrillation, atrial arrhythmias, GLP-1 receptor agonists, obesity, narrative review

## Abstract

Cardiac arrhythmias, particularly atrial fibrillation (AF), represent a major cause of morbidity and mortality worldwide, especially in individuals with type 2 diabetes mellitus (T2D), obesity, and cardiovascular disease. Traditional antiarrhythmic drugs (AADs) remain the cornerstone of rhythm control, but their efficacy is limited and often accompanied by adverse effects. A multidimensional approach that includes upstream therapy—targeting metabolic, structural and inflammatory substrates—has therefore emerged as a critical strategy in arrhythmia prevention and management. Among novel therapeutic agents, glucagon-like peptide-1 receptor agonists (GLP-1 RAs), particularly semaglutide, are gaining attention for their potential antiarrhythmic properties beyond glycaemic control. This review discusses multidimensional arrhythmia management, current indications for semaglutide, and future perspectives on its role as a potential upstream antiarrhythmic therapy.

## 1. Introduction

Atrial fibrillation (AF) and atrial flutter (AFl) are common supraventricular arrhythmias [1,2,3]. These arrhythmias are associated with increased morbidity and mortality, leading to complications such as systemic embolic events, stroke, and heart failure (HF) (tachycardia-induced cardiomyopathy) [2,3]. Due to their prevalence and clinical significance, there have been numerous diagnostic and therapeutic advances over the decades in the management of these arrhythmias. Current European guidelines recommend an integrated approach that includes rate control, rhythm control, prevention of thromboembolic complications (through a careful assessment of the risk-benefit ratio), and management of the patient’s comorbidities, as well as a shared decision with the patient regarding the therapeutic approach [3]. In the management of comorbidities (particularly diabetes and obesity), drugs belonging to the GLP-1 receptor agonist (GLP-1 RA) class—and semaglutide in particular—are becoming increasingly important. Beyond their well-established glucose-lowering and weight-reducing effects, GLP-1 RAs have demonstrated broad cardiovascular benefits, including a reduction in major adverse cardiovascular events (MACE) in selected high-risk populations [4,5]. These benefits may be particularly relevant in patients with AF and AFl, given the close relationship between supraventricular arrhythmias and cardiometabolic risk factors such as obesity, diabetes, hypertension, and systemic inflammation [6,7]. Semaglutide, through its favourable effects on body weight, glycaemic control, blood pressure, inflammatory pathways, and potentially cardiac structure and function, may therefore influence both the substrate and the clinical consequences of these arrhythmias [8,9]. Moreover, emerging clinical evidence suggests a possible association between GLP-1 RA treatment and a lower occurrence of AF in semaglutide-treated patients [10]. These observations raise the possibility that semaglutide could provide benefits extending beyond conventional metabolic control and contribute to a more comprehensive management of patients with supraventricular arrhythmias. The purpose of this narrative review is to evaluate the potential role of semaglutide as a drug capable of improving the management and prognosis of patients with supraventricular arrhythmias, through a comprehensive review of the currently available scientific evidence.

## 2. A Multidimensional Approach to Patients with Atrial Arrhythmia

The management of cardiac arrhythmias has historically revolved around the use of dedicated antiarrhythmic drugs (AADs), catheter ablation, and device-based therapies [11]. The limitations of AADs—modest efficacy, proarrhythmic potential, and systemic side effects—have led clinicians and researchers to recognise the need for a broader, multidimensional approach. This strategy extends beyond rhythm control alone and addresses upstream risk factors, including metabolic dysfunction, inflammation, structural remodelling, and autonomic imbalance [3,11,12,13,14].

Renin-angiotensin-aldosterone system inhibition illustrates the concept of upstream therapy: by reducing pressure and volume load and attenuating profibrotic signalling, these agents may reduce incident AF in selected patients with hypertension or HF. The effect is context-dependent and does not justify their use solely for rhythm prevention [3,11,12].

Other pharmacological classes provide similar, although heterogeneous, examples. Beta-blockers directly influence sympathetic triggers and remain conventional rate- and rhythm-modifying agents, whereas evidence for statins and mineralocorticoid receptor antagonists is largely restricted to specific clinical settings. These observations support substrate-directed management but should not be interpreted as uniform antiarrhythmic class effects [3,11,13].

Sodium-glucose cotransporter 2 (SGLT2) inhibitors provide a more contemporary example of cardiometabolic upstream therapy. Their established benefits in HF are accompanied by signals of lower atrial arrhythmia risk in meta-analyses and device-monitored cohorts, plausibly mediated by haemodynamic unloading, improved energetics, and reduced oxidative stress [15,16,17].

More recently, vericiguat, a soluble guanylate cyclase stimulator used in selected patients with heart failure with reduced ejection fraction (HFrEF), has also been associated with a reduction in device-detected atrial and ventricular arrhythmic events in a small retrospective cohort of implantable cardioverter-defibrillator/cardiac resynchronisation therapy-defibrillator (ICD/CRT-D) carriers. This finding should be considered hypothesis-generating and requires confirmation in larger prospective studies [18].

Collectively, these examples support the paradigm that effective arrhythmia management cannot rely solely on AADs or interventional therapies. Instead, targeting upstream modulators of cardiac remodelling and systemic metabolic dysfunction is essential for comprehensive care. Within this framework, semaglutide and other GLP-1 RAs represent a promising frontier, given their proven cardiometabolic benefits and emerging evidence of antiarrhythmic effects [3,5,13,19].

The AF-CARE framework formalises this multidimensional approach by placing comorbidity and risk-factor management alongside stroke prevention and symptom-directed rate or rhythm control. In this context, semaglutide should be evaluated as a substrate-modifying therapy rather than as a replacement for antiarrhythmic drugs or catheter ablation [3].

## 3. Current Indications and Cardiovascular Evidence for Semaglutide

Semaglutide has rapidly evolved from a glucose-lowering agent to a cornerstone therapy in cardiometabolic medicine, with current indications that span type 2 diabetes mellitus (T2D), obesity, and cardiovascular prevention [5,20]. Initially developed as a potent GLP-1 RA, its first clinical application was in the management of T2D, where it improves glycaemic control by enhancing glucose-dependent insulin secretion, suppressing glucagon release, and slowing gastric emptying. The SUSTAIN trial programme and cardiovascular outcome trials provided foundational evidence for glycaemic efficacy and cardiovascular safety, while STEP trials established its efficacy for chronic weight management [21,22,23,24].

The therapeutic profile of semaglutide soon expanded into the cardiovascular domain. The SUSTAIN-6 trial demonstrated that once-weekly subcutaneous semaglutide was associated with a 26% reduction in MACE in patients with T2D and high cardiovascular risk (HR 0.74, 95% CI 0.58–0.95), providing evidence of cardiovascular protection beyond glucose lowering [21]. PIONEER 6 established cardiovascular safety for oral semaglutide [20]. More recently, the SOUL trial extended this evidence base, enrolling over 9600 patients with T2D and established atherosclerotic cardiovascular disease (CVD) or chronic kidney disease; oral semaglutide reduced cardiovascular death, myocardial infarction, or stroke by 14% compared with placebo [25].

Beyond diabetes and cardiovascular protection, semaglutide has transformed the pharmacological management of obesity. STEP 1 showed a mean body-weight reduction of approximately 15% over 68 weeks with once-weekly semaglutide 2.4 mg in adults with overweight or obesity without diabetes [22]. STEP 2 confirmed efficacy in patients with obesity and T2D, and STEP 4 showed that continued treatment maintained weight loss [23,24]. Furthermore, in addition to its effects on body weight, semaglutide may also influence the endocrine and inflammatory function of adipose tissue. A proteomic analysis of samples from the STEP 1 and STEP 2 trials identified treatment-related changes in circulating adipokines, including adiponectin and leptin, together with broader changes in proteins involved in lipid metabolism and inflammatory pathways. This observation underlines the importance of this drug in metabolic and inflammatory profiles, with a possible beneficial effect on arrhythmic burden [26]. In addition, the SELECT trial showed that semaglutide 2.4 mg reduced cardiovascular events by 20% in adults with overweight or obesity, established CVD, and no diabetes [5].

The evolution of clinical guidelines reflects this growing evidence. The ADA Standards of Care and the 2023 ESC Guidelines on CVD in diabetes recommend GLP-1 RAs with proven cardiovascular benefit, including semaglutide, in patients with T2D and established or high-risk atherosclerotic CVD, while chronic weight-management indications derive from the STEP and SELECT evidence base [27,28].

Although no current guideline explicitly lists arrhythmia prevention among the approved indications for semaglutide, its ability to modify upstream risk factors such as obesity, inflammation, and structural heart disease situates it as a relevant therapy within a multidimensional arrhythmia management framework [3,11,13,19,29,30,31].

## 4. Mechanisms Potentially Linking Semaglutide to Arrhythmia Reduction

The potential antiarrhythmic effect of semaglutide is best understood within the paradigm of upstream therapy, targeting the systemic and cardiac substrates that give rise to arrhythmias rather than the arrhythmias themselves [3,11]. Unlike conventional antiarrhythmic drugs, which primarily act on ion channels to suppress abnormal electrical activity, semaglutide influences a broad range of pathophysiological processes implicated in arrhythmogenesis, thereby offering the possibility of modifying disease progression in a multidimensional way [5,22,29,30,32,33].

One potentially relevant mechanism is the reduction of epicardial and visceral adipose tissue. Epicardial fat lies in close anatomical and paracrine proximity to the atrial myocardium and releases inflammatory and profibrotic mediators that may contribute to conduction abnormalities and atrial fibrosis [34,35,36,37]. Excess visceral adiposity also exerts systemic metabolic stress, further worsening the arrhythmic substrate. Greater epicardial fat volume has been associated with AF recurrence after catheter ablation [38]. The potential to modify adipose tissue biology is supported by human tissue studies linking epicardial GLP-1 receptor expression to genes involved in fatty acid oxidation and white-to-brown fat differentiation, although these associations do not establish a treatment effect [39].

Semaglutide produces substantial and sustained weight loss, and small GLP-1 RA imaging studies, including semaglutide data from STOP, suggest reductions in epicardial or visceral fat depots [40,41,42]. However, much of the evidence linking these changes to AF substrate modification remains indirect [43]. Changes in left atrial structure in STEP-HFpEF provide separate evidence of a potentially favourable substrate effect, without establishing that it is mediated by epicardial fat loss [44]. The contribution of overall weight loss therefore remains difficult to distinguish from a direct local myocardial or adipose-tissue effect.

Inflammation represents a second important target. Chronic low-grade inflammation is a hallmark of diabetes and obesity and contributes to atrial and ventricular remodelling through fibrosis, oxidative stress, and impaired calcium handling [34]. Semaglutide has been associated with reductions in inflammatory biomarkers, including C-reactive protein, in STEP-HFpEF [29,30]. Proteomic analyses of STEP 1 and STEP 2 also identified changes in adipokines and other inflammatory and metabolic proteins [26]. Together, these findings support a potentially less arrhythmogenic substrate.

A third potential pathway through which GLP-1RAs might exert a beneficial effect involves modulation of the renin–angiotensin–aldosterone system (RAAS), given the contribution of aldosterone signalling to atrial remodelling [45]. Experimental work with liraglutide observed a reduction in aldosterone secretion mediated by increased production of atrial natriuretic peptide (ANP) mediated by GLP-1Ras [46]. Furthermore, a direct clinical evidence comes from a double-blind trial of 60 participants with T2D and albuminuria receiving empagliflozin and background renin–angiotensin system inhibition. Adding semaglutide, titrated to 1.0 mg weekly over a 26-week treatment period, reduced plasma aldosterone compared with placebo, without a significant change in plasma renin. However, aldosterone was a secondary endpoint, and the analysis included only 33 participants because many measurements were below the assay detection limit [47]. This finding suggests a possible effect on aldosterone but does not demonstrate broad RAAS suppression or establish that this pathway contributes to AF prevention. Nevertheless, the link between RAAS inhibition, ANP, atrial remodelling and AF remains of interest, and future studies should explore more deeply the possible implications of this axis.

A fourth dimension involves autonomic regulation. GLP-1 RAs commonly increase resting heart rate, although the magnitude and duration of this effect vary among individual agents and tend to be greater with long-acting compounds [48]. With semaglutide, the increase is generally modest: in SUSTAIN-6, mean pulse rate increased by 2.1 and 2.4 beats per minute from baseline with the 0.5- and 1.0-mg once-weekly doses, respectively [21]. This effect may reflect a combination of direct sinoatrial stimulation and changes in autonomic regulation, although its precise mechanism in humans remains uncertain [48].

The modest increase in heart rate may appear to contrast with the proposed antiarrhythmic effects of semaglutide. Nevertheless, pooled randomised evidence for GLP-1RAs does not show a significant overall increase in atrial or ventricular arrhythmias [49], and semaglutide-specific meta-analyses suggest fewer reported AF events [10,19,50]. These data suggest that the benefits reported in the studies may be attributable to other mechanisms (improvements in weight, adipose-tissue biology, inflammation, and atrial loading) rather than a possible effect on the autonomic nervous system [26,30,42,44]. Nevertheless, given the importance of the autonomic nervous system on heart rate and arrhythmias, the possibility that the pleiotropic effects of GLP-1RAs include modulation of the autonomic nervous system (with potential protective effects against AF and other arrhythmias) appears to be of interest. Therefore, since most trials captured arrhythmias as secondary or adverse-event outcomes, dedicated rhythm studies are needed to establish the net effect in humans.

Finally, semaglutide appears to exert beneficial effects on myocardial structure and function. In patients with T2D or obesity, the myocardium often exhibits steatosis, diastolic dysfunction, and interstitial fibrosis, all of which promote electrical instability. STEP-HFpEF analyses showed improvements in symptoms, inflammation, and cardiac remodelling, supporting a plausible substrate-modifying effect, although AF burden was not the primary endpoint [29,30,31,44].

Taken together, these mechanisms illustrate how semaglutide, through its systemic metabolic benefits, produces downstream cardioprotective effects that intersect with arrhythmia pathophysiology [22,26,29,30,44]. Rather than exerting a direct antiarrhythmic action, semaglutide modifies the environment in which arrhythmias occur—reducing triggers, stabilising conduction, and reversing structural abnormalities. While definitive arrhythmia-focused trials are still needed, the convergence of mechanistic insights provides a strong rationale for investigating semaglutide as part of a multidimensional strategy for arrhythmia prevention [3,11,19].

## 5. Evidence from Observational Studies, Randomised Studies and Meta-Analyses

A prespecified echocardiographic analysis by Solomon et al. evaluated 491 participants from the STEP-HFpEF programme. Semaglutide attenuated the increase in left atrial volume over 52 weeks compared with placebo (estimated treatment difference −6.13 mL, 95% CI −9.85 to −2.41; *p* = 0.0013), without a significant effect on left ventricular mass or systolic function [44]. This supports a potentially favourable effect on the atrial substrate, but AF burden was not measured.

A post hoc analysis of the global, randomised, phase 3 SUSTAIN 6 and PIONEER 6 trials showed that the risk of any stroke was not lower in the semaglutide group than in the placebo group among patients with prior AF, although the number of events was very low in this subgroup (7 versus 1 events) [51]. This was an exploratory post hoc analysis with few participants and a limited number of events within the AF subgroup, and it did not consider transient ischaemic attacks or secondary stroke prevention [51].

A retrospective study by Baser et al. examined the association between newly approved anti-obesity medications (semaglutide and tirzepatide) and cardiovascular events in 5926 patients with obesity. The proportion of any CVD outcome was significantly lower in the GLP-1 RA-treated cohort than in controls (19.36% vs. 21.16%, *p* = 0.0010), as were the proportions of HF (4.89% vs. 6.13%, *p* = 0.0001), AF (3.83% vs. 5.17%, *p* < 0.0001), any arrhythmia (3.49% vs. 4.14%, *p* = 0.0153), and peripheral vascular disease (2.94% vs. 3.44%, *p* = 0.0395); only ischaemic heart disease was more frequent in the GLP-1 RA cohort (0.34% vs. 0.20%, *p* = 0.0289). Coronary artery disease (CAD) and stroke did not differ significantly between groups. GLP-1 RA use among patients with obesity was associated with an 8% lower risk of CVD than in controls (hazard ratio [HR] = 0.92, 95% CI 0.86–0.98; *p* = 0.0068) in a Cox regression analysis. Overall, semaglutide and tirzepatide were associated with a lower incidence of AF and arrhythmias than no use of these drugs [52].

Catheter ablation for AF has a higher failure rate in patients with obesity, whereas success rates improve when patients achieve goal-oriented weight reduction before ablation [53,54]. Nevertheless, diet- and exercise-based weight-loss strategies may have limited efficacy and may delay ablation, reducing the likelihood of maintaining or restoring sinus rhythm [55,56]. In this setting, GLP-1 RAs could provide weight loss over a shorter period and may represent a potential bridging therapy before catheter ablation. A prospective single-centre pilot study aims to evaluate the efficacy of daily liraglutide injections in patients with obesity (body mass index [BMI] ≥30 kg/m^2^) and symptomatic AF for at least 13 weeks before and 52 weeks after AF ablation. This ongoing study includes exploratory outcomes assessing changes in atrial structure, function, and fibrosis by cardiac magnetic resonance imaging, electroanatomic mapping, and patient-reported outcome measures [57].

Several systematic reviews and meta-analyses have evaluated GLP-1 RAs and AF/AFl. Despite an overall neutral class effect on arrhythmia risk, data on semaglutide suggest a possible drug-specific benefit in reducing AF occurrence [50,58,59,60,61].

Saglietto et al. included 10 randomised controlled trials (RCTs) and 12,651 participants at high cardiovascular risk, mainly with T2D, and reported a 42% relative reduction in reported AF with semaglutide (RR 0.58, 95% CI 0.40–0.85; I^2^ = 0%), without a statistically significant interaction by oral versus subcutaneous administration [50].

A broader safety meta-analysis of the SUSTAIN and PIONEER programmes reported a 31% reduction in AF with semaglutide (relative risk [RR] 0.69, 95% CI 0.50–0.95; *p* = 0.025; I^2^ = 0%) [58].

GLP-1 RA treatment is associated with an increase in heart rate, which could potentially induce cardiac arrhythmias. Wu et al. pooled 56 RCTs involving 79,720 participants with T2D or obesity. GLP-1 RAs did not significantly alter the overall risk of AF (RR 0.97, 95% CI 0.83–1.12), AFl, ventricular arrhythmias (RR 1.24, 95% CI 0.92–1.67), or sudden cardiac death. Drug-specific and high-dose subgroup findings were exploratory and sensitive to the inclusion of individual large trials [49]. Zhang et al. included 21 semaglutide RCTs with 25,957 participants and reported a lower risk of AF overall (RR 0.70, 95% CI 0.52–0.95). The placebo-only subgroup did not reach statistical significance (RR 0.77, 95% CI 0.56–1.07), indicating that comparator choice and the trials included affect the pooled estimate [10].

Finally, an updated meta-analysis of 26 RCTs involving 48,583 participants (25,879 treated with semaglutide) and 541 new-onset AF events showed lower odds of new-onset AF compared with control treatment (odds ratio [OR] 0.83, 95% CI 0.70–0.98; *p* = 0.03; I^2^ = 0%). The effect was more pronounced with oral semaglutide (OR 0.48, 95% CI 0.24–0.95; *p* = 0.04) and in active-comparator trials (OR 0.41, 95% CI 0.20–0.83; *p* = 0.01). In trials without concomitant SGLT2 inhibitor therapy, new-onset AF was reduced by 21% (OR 0.79, 95% CI 0.63–0.99; *p* = 0.04) [19].

Guo et al. reported in a prospective, non-randomised study in 2025 that semaglutide treatment following catheter ablation was associated in 437 consecutive patients with T2D and a BMI ≥24 kg/m^2^ with a lower 12-month recurrence of atrial arrhythmias (HR 0.68, 95% CI 0.49–0.95; *p* = 0.030) [62]. In 2026, Ciconte et al. extended this observation to a predominantly non-diabetic obese population monitored with implantable cardiac monitors during a median follow-up of 18 months (freedom from recurrence 80.2% vs. 65.2%; HR 0.52, 95% CI 0.34–0.78; *p* = 0.002) [63]. Continuous rhythm surveillance strengthens endpoint ascertainment, but non-randomised allocation and residual confounding preclude causal inference.

Across meta-analyses, the apparent reduction in reported AF varies across analyses and is based on relatively few events that were commonly collected as adverse-event outcomes [50,58,59,60,61].

## 6. Comparison with Other GLP-1 Receptor Agonists and Other Antidiabetic Drugs

A post hoc analysis of data from the REWIND trial assessed the occurrence of AF or AFl on annual ECG in 9543 patients with T2D treated with once-weekly subcutaneous dulaglutide versus placebo. Of these patients, 524 (5.5%) had at least one episode of AF/AFl during a median of 5.4 years of follow-up. A total of 4769 patients were randomised to dulaglutide, and 269 (5.6%) had AF/AFl, whereas 255 (5.3%) of the 4774 patients allocated to placebo had AF/AFl. Therefore, dulaglutide did not reduce the incidence of AF/AFl in this population [64,65].

A subanalysis from the multicentre, event-driven, double-blind, placebo-controlled Harmony Outcomes trial compared the effects of albiglutide versus placebo on AF occurrence in 9463 patients with T2D and established CVD. Albiglutide was associated with numerically fewer AF events than placebo (108 vs. 131), irrespective of baseline history of AF, although this observation did not reach statistical significance [66].

A study using data from a multicentre healthcare provider in Taiwan enrolled 16,566 patients with T2D treated with SGLT2 inhibitors (empagliflozin, dapagliflozin, and canagliflozin) and 2746 patients treated with GLP-1 RAs (liraglutide and dulaglutide). In this real-world setting, SGLT2 inhibitors were associated with a lower risk of new-onset AF than GLP-1 RAs in patients with T2D [67].

An analysis of the FAERS database of reported adverse events, including AF, with GLP-1 RAs, DPP-4 inhibitors, and SGLT2 inhibitors in patients with T2D found that linagliptin had the highest proportion of AF events, followed by saxagliptin, liraglutide, sitagliptin, semaglutide, and exenatide [68]. These differences suggest the cardiac safety of GLP-1 RAs and a potential AF reporting signal for some DPP-4 inhibitors, but should be interpreted cautiously because of differences in drug exposure, concomitant treatment, and reporting bias.

Another study compared the incidence of new-onset AF among older adults (aged ≥66 years) with T2D after starting therapy with an SGLT2 inhibitor, a DPP-4 inhibitor, or a GLP-1 RA in routine clinical practice. The risk of incident AF was lower in the SGLT2 inhibitor group than in the matched DPP-4 inhibitor or GLP-1 RA groups [69].

Despite their modest chronotropic effect, GLP-1 RAs have not demonstrated a significant overall increase in atrial or ventricular arrhythmias in pooled randomised evidence. Molecule-specific and high-dose subgroup signals remain exploratory and do not establish uniform safety or benefit across all patients [49].

A meta-analysis of three propensity-matched studies involving 6031 participants found that GLP-1 RA use after AF ablation was associated with lower recurrence over 12 months (HR 0.549, 95% CI 0.315–0.956; *p* = 0.034; I^2^ = 57%) [70]. The pooled estimate is based on observational, class-level evidence and cannot establish a semaglutide-specific causal effect.

In addition, an umbrella review by Zhu et al. of 232 systematic reviews and meta-analyses of RCTs examining the cardiovascular safety of ten classes of glucose-lowering medications included AF among the prespecified cardiovascular outcomes assessed. However, the main protective associations identified for GLP-1 RAs as a class concerned MACE, cardiovascular death, myocardial infarction, stroke, and HF, rather than AF. In molecule-specific analyses, albiglutide reduced MACE, myocardial infarction, and HF; dulaglutide reduced stroke; exenatide and liraglutide reduced MACE; and semaglutide reduced MACE and stroke. These findings provide broader cardiometabolic context but do not establish a semaglutide-specific antiarrhythmic effect [71] (Table 1).

Taken together, these data do not establish a uniform antiarrhythmic class effect. Differences between molecules, formulations, populations, and methods of arrhythmia ascertainment may explain discordant estimates. Semaglutide currently has the most coherent drug-specific signal, but no head-to-head randomised trial has demonstrated superiority for AF prevention. Figure 1 depicts the mechanistic pathways potentially linking semaglutide to modification of the atrial arrhythmogenic substrate, whereas Figure 2 shows the hierarchy of current evidence on semaglutide and its effects on AF.

## 7. Ongoing Trials Regarding GLP-1 RAs and AF/AFl

A search of ClinicalTrials.gov identified prospective studies of semaglutide and AF/AFl. DUTCH-WAIST (NCT06184633; EUCT 2022–501392–81–00) is a multicentre, double-blind, randomised, placebo-controlled trial of once-weekly subcutaneous semaglutide 2.4 mg versus placebo designed to quantify the effect of semaglutide-induced weight loss on rhythm control through reversal of atrial remodelling at 12 months [72,73]. The updated Dutch registry lists recruitment as stopped and includes both a hierarchical rhythm-control outcome and a binary clinical composite as primary endpoints [73].

SEMINAL-AF (NCT06499857) is a recruiting phase 3 randomised trial assessing whether semaglutide-related weight loss affects AF severity, AF burden, cardiac remodelling, and recurrence risk, assessed by routine outpatient rhythm monitoring [74]. The rationale is supported by the established association between obesity and incident, postoperative, and post-ablation AF [75].

For liraglutide, the GOAL-AF protocol (NCT05221229) evaluates liraglutide-supported weight loss before and after AF ablation, whereas the recently published LEAF trial (NCT03856632) evaluated adjunctive liraglutide in overweight or obese patients with paroxysmal or persistent AF undergoing ablation, assessing epicardial adipose tissue (EAT) and AF/atrial flutter recurrence [57,76,77]. One-year freedom from AF/AFl, a secondary endpoint, was 81% with liraglutide-supported risk-factor modification versus 54% with risk-factor modification alone (log-rank *p* = 0.007) [77].

No additional AF-focused studies were identified in the same registry search for other GLP-1 RAs, including exenatide, lixisenatide, dulaglutide, or albiglutide, or for related incretin-based therapies such as tirzepatide.

The WAIT trial (NCT07275697), registered in 2025, was designed as a randomised phase 4 trial evaluating whether pre-ablation metabolic optimisation with once-weekly semaglutide 2.4 mg could improve rhythm outcomes in patients with overweight or obesity undergoing a first AF catheter ablation. The primary endpoint is arrhythmia-free survival at 12 months after ablation, with recurrence defined as implantable-loop-recorder-detected AF, AFl, or atrial tachycardia lasting at least 30 s after a 3-month blanking period [78].

SOCRATES-AF (NCT04885634) was designed as a prospective, parallel-group, double-blind, randomised, placebo-controlled pilot trial to assess the feasibility of semaglutide 2.4 mg once weekly, in addition to standard AF care, for reducing arrhythmia burden in overweight or obese patients with symptomatic paroxysmal or early persistent AF, with recurrence assessed using implantable cardiac monitoring [79]. However, the registry currently lists the study as withdrawn, and no participants were enrolled or outcome data reported [79].

Together with the suspended trial NCT05209165, DUTCH-WAIST (NCT06184633), SEMINAL-AF (NCT06499857), and WAIT (NCT07275697) reflect a shift from opportunistic adverse-event analyses towards prospectively measured AF burden and recurrence [72,73,74,78,80]. Table 2A–D and Table 3 summarise the main characteristics of the key studies included in this narrative review.

## 8. Conclusions

Semaglutide is emerging as a potentially relevant component of the multidimensional management of patients with AF or AFl. Its principal value lies in modifying the cardiometabolic and structural substrate that promotes atrial arrhythmogenesis—including obesity, systemic inflammation, epicardial adiposity, and HF—rather than in exerting the direct ion-channel effects of a conventional antiarrhythmic drug [3,13,26,29,30,31,42,44,81]. This distinction is clinically important: semaglutide may complement, but cannot replace, established rhythm-control strategies [3].

Current randomised-trial meta-analyses suggest a reduction in reported AF events, while emerging observational studies indicate a possible reduction in recurrence after catheter ablation, particularly in patients with obesity. However, the limited number of events, heterogeneous rhythm ascertainment, overlap between trial populations, and residual confounding prevent definitive causal conclusions. At present, semaglutide should therefore be prescribed according to established metabolic and cardiovascular indications; a potential arrhythmic benefit may represent an additional advantage in appropriately selected patients. Dedicated randomised trials incorporating continuous or systematic rhythm monitoring are required to determine whether semaglutide produces clinically meaningful reductions in AF burden and recurrence, and whether these effects extend beyond weight loss and cardiometabolic improvement [10,19,50,58,62,63,70,72,74,77,78,80].

## Figures and Tables

**Figure 1:**
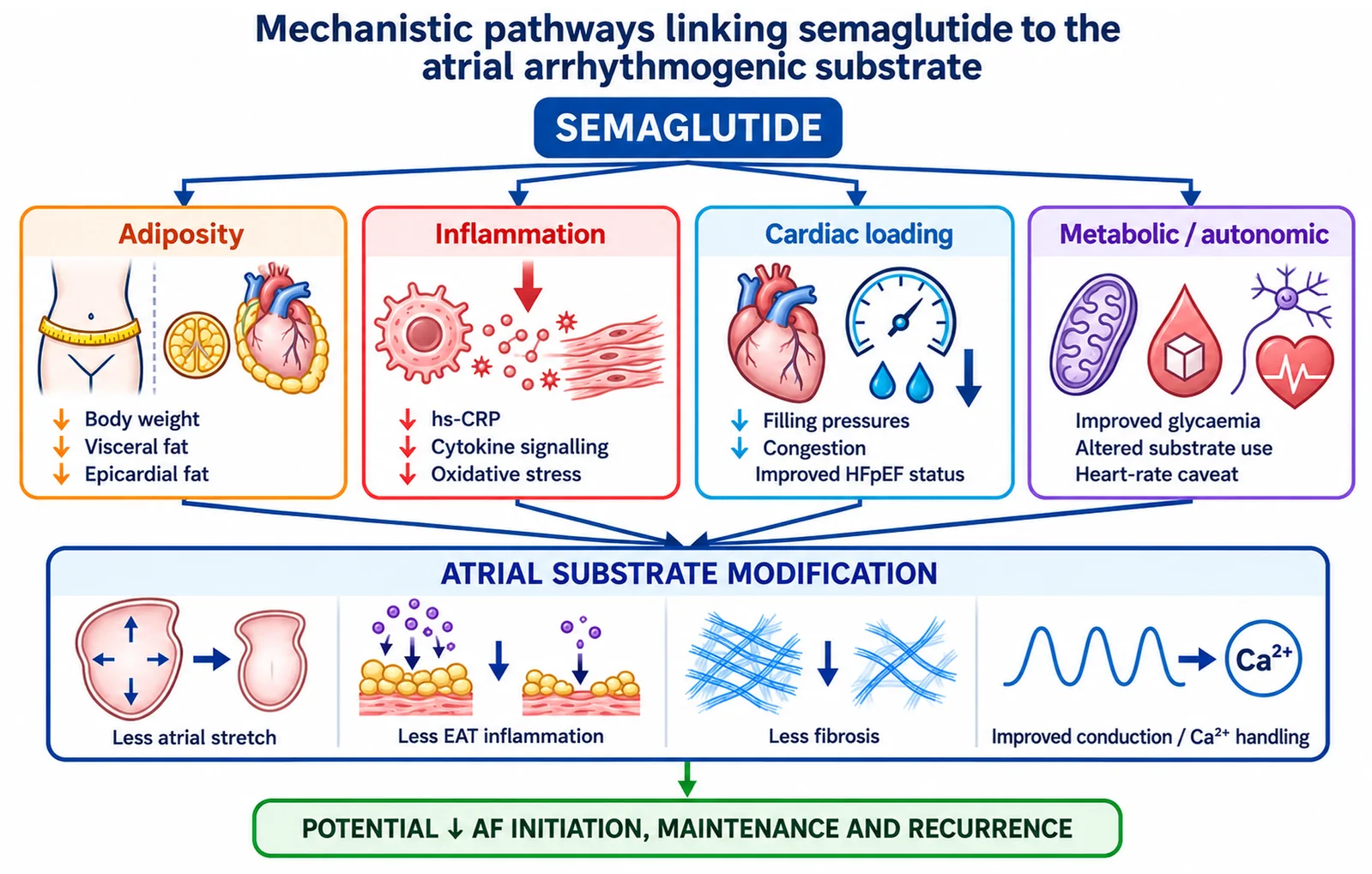
Mechanistic pathways potentially linking semaglutide to modification of the atrial arrhythmogenic substrate. Abbreviations: AF, atrial fibrillation; HFpEF, heart failure with preserved ejection fraction; hs-CRP, high-sensitivity C-reactive protein.

**Figure 2:**
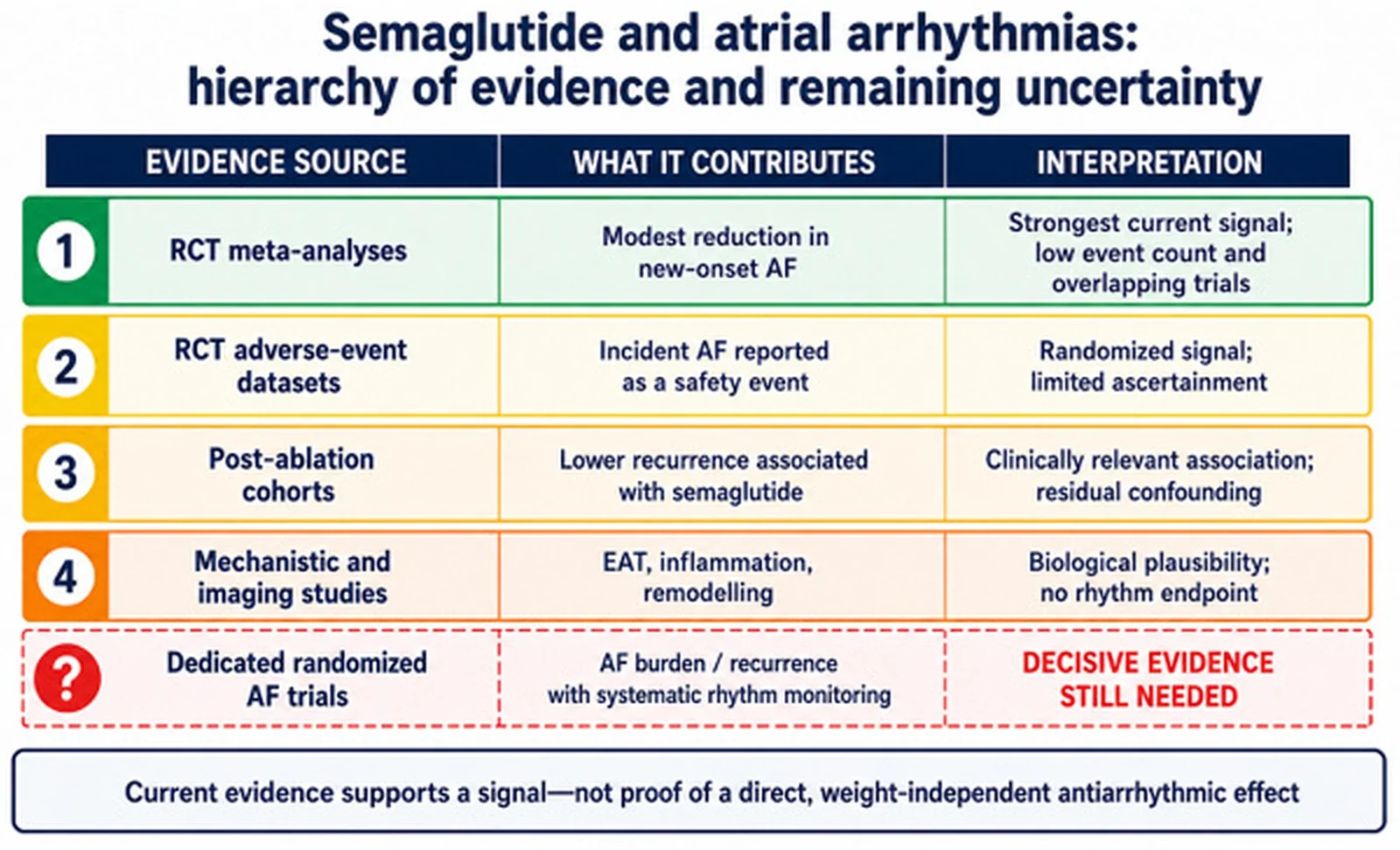
Hierarchy of current evidence and principal sources of uncertainty. Abbreviations: EAT, epicardial adipose tissue; RCT, randomised controlled trial.

**Table 1:** Cardiovascular outcomes associated with GLP-1 receptor agonists in the umbrella review by Zhu et al. [71].

GLP-1 RA/Analysis Level	MACE	CV Death	MI	HF	Stroke
GLP-1 RAs as class	↓	↓	↓	↓	↓
Semaglutide	↓	-	-	-	↓
Albiglutide	↓	-	↓	↓	-
Dulaglutide	-	-	-	-	↓
Exenatide	↓	-	-	-	-
Liraglutide	↓	-	-	-	-

A downward arrow indicates a reported reduction in the specified cardiovascular outcome in the umbrella-review evidence by Zhu et al. [71]; “-” indicates that no clear reduction was reported for that molecule/outcome in the summarised evidence, not necessarily the absence of effect. AF was included among the cardiovascular outcomes assessed in the umbrella review, but the main protective associations identified for GLP-1 RAs concerned MACE, cardiovascular death, myocardial infarction, stroke, and HF rather than AF. CV, cardiovascular; HF, heart failure; MACE, major adverse cardiovascular events; MI, myocardial infarction.

**Table 2:** Clinical evidence concerning GLP-1 receptor agonists and atrial arrhythmia outcomes, stratified according to outcome and method of ascertainment. (**A**) Atrial fibrillation occurrence in randomised trials and pooled analyses. (**B**) Atrial arrhythmia recurrence after catheter ablation. (**C**) Structural and mechanistic outcomes. (**D**) Comparative and real-world evidence.

**(A)**										
**Study**	**Design and Population**	**Exposure**		**Endpoint Status and Ascertainment**			**Events and Absolute Risk**		**Effect Estimate**	**Main Limitation**
Cesaro et al., 2025 [19]	Updated systematic review and meta-regression; 26 RCTs; 48,583 participants, including 25,879 treated with semaglutide	Semaglutide vs. placebo or active comparator		AF generally not prespecified in the original trials; predominantly derived from adverse-event reporting without systematic rhythm monitoring			541 reported AF events; first-ever incident AF not uniformly separable		OR 0.83, 95% CI 0.70–0.98; *p* = 0.03; I^2^ = 0%	Low event rate; overlapping trial populations; heterogeneous and potentially incomplete ascertainment; subgroup analyses exploratory
Zhang et al., 2024 [10]	Systematic review and meta-analysis; 21 RCTs; 25,957 participants	Semaglutide vs. placebo or active comparator		Reported AF; generally a secondary or safety outcome			Arm-specific absolute events NR		RR 0.70 (95% CI 0.52–0.95); placebo-only RR 0.77 (0.56–1.07)	Sparse events; overlapping trial populations; placebo-only estimate not significant
Saglietto et al., 2024 [50]	Systematic review and meta-analysis; 10 RCTs; 12,651 high-CV-risk participants	Semaglutide vs. placebo		Secondary/safety outcome; AF generally collected as an adverse event			Arm-specific events NR; pooled control incidence 0.86 events per 100 person-years		RR 0.58, 95% CI 0.40–0.85; I^2^ = 0%	Few AF events; no dedicated rhythm monitoring; substantial overlap with other meta-analyses
Yin et al., 2021 [58]	Safety meta-analysis of SUSTAIN and PIONEER trials	Semaglutide vs. control		Safety outcome derived from reported adverse events; not a prespecified rhythm endpoint			NR		RR 0.69, 95% CI 0.50–0.95; *p* = 0.025; I^2^ = 0%	Safety meta-analysis; incomplete rhythm ascertainment; overlapping trial datasets
Wu et al., 2022 [49]	Systematic review and meta-analysis; 56 RCTs; 79,720 participants with T2D or obesity	GLP-1 RAs vs. placebo or control		Arrhythmias mainly extracted from adverse-event reporting; no uniform adjudication or monitoring			NR		AF: RR 0.97 (95% CI 0.83–1.12); ventricular arrhythmias: RR 1.24 (0.92–1.67)	Class-level analysis; heterogeneous
Monami et al., 2017 [59]	Systematic review; 113 eligible RCTs; 31 trials with AF events, 33,271 participants	GLP-1 RAs vs. comparators		AF reported as a safety/adverse-event outcome			NR		OR 0.87, 95% CI 0.71–1.05; *p* = 0.15	Older evidence base; sparse events; no systematic rhythm monitoring
Wei et al., 2022 [60]	Meta-analysis of 8 cardiovascular-outcome trials; 60,081 participants with T2D	GLP-1 RAs vs. controls		Arrhythmia outcomes extracted from randomised-trial safety data			NR		AF: RR 0.96, 95% CI 0.86–1.07; *p* = 0.43	Heterogeneous populations, drugs, comparators, and outcome definitions
Hamedi et al., 2021 [61]	Review/meta-analysis of GLP-1 RA cardiovascular-outcome trials	GLP-1 RAs vs. placebo		AF generally identified as an adverse event rather than a prespecified endpoint			NR		No clear excess AF risk for the GLP-1 RA class	Limited sensitivity for paroxysmal or asymptomatic AF
REWIND post hoc, 2022 [64,65]	Post hoc analysis of randomised REWIND trial; 9543 participants without baseline atrial arrhythmia; median follow-up 5.4 years	Dulaglutide vs. placebo		Post hoc AF/AFl endpoint assessed by annual 12-lead ECG; not continuous monitoring			269/4769 (5.6%) vs. 255/4774 (5.3%)		No significant between-group difference	Annual ECG substantially underdetects paroxysmal and asymptomatic AF
Harmony Outcomes post hoc, 2026 [66]	Post hoc analysis of 9463 participants with T2D and established CVD	Albiglutide vs. placebo		Post hoc AF outcome; no dedicated continuous rhythm monitoring			108 vs. 131 AF events; 239/9463 overall (2.5%)		HR 0.82, 95% CI 0.63–1.06; *p* = 0.12	Not powered for AF; numerically favourable but statistically non-significant
(**B**)										
**Study**	**Design and Population**	**Exposure**		**Endpoint Status and Ascertainment**			**Events and Absolute Risk**		**Effect Estimate**	**Main Limitation**
Guo et al., 2025 [62]	Prospective, non-randomised cohort; T2D, BMI ≥24 kg/m^2^, first AF ablation; 158 semaglutide-treated and 279 untreated participants	Post-ablation semaglutide vs. no semaglutide		Recurrence after a 3-month blanking period; scheduled follow-up at 1, 3, 6, 9 and 12 months			Arm-specific absolute events NR		HR 0.68, 95% CI 0.49–0.95; *p* = 0.030	Non-randomised allocation; residual confounding; potential mediation by weight loss and improved glycaemic control
Ciconte et al., 2026 [63]	Single-centre propensity-matched cohort; obesity and paroxysmal AF; first ablation; 181 treated patients and 181 controls	Semaglutide initiated within 3 months before or 1 month after ablation		Primary study endpoint: AF, AFl, or atrial tachycardia ≥30 s beyond a 2-month blanking period; continuous implantable cardiac monitoring			Recurrence: 19.8% vs. 34.8%; freedom from recurrence: 80.2% vs. 65.2% at 18 months		HR 0.52, 95% CI 0.34–0.78; *p* = 0.002	Observational design; single centre; substantially greater weight loss with semaglutide; residual confounding
Karakasis et al., 2024 [70]	Meta-analysis of 3 propensity-matched studies; 6031 participants	GLP-1 RAs vs. no GLP-1 RA after ablation		Recurrence definitions and monitoring strategies varied across included studies			NR		HR 0.549, 95% CI 0.315–0.956; *p* = 0.034; I^2^ = 57%	Only 3 observational studies; mixed GLP-1 RA molecules; moderate heterogeneity and residual confounding
LEAF, 2026 [77]	Randomised trial; 59 participants with BMI ≥27 kg/m^2^; 47 (80%) had persistent AF	Risk-factor modification plus liraglutide (*n* = 31) vs. risk-factor modification alone (*n* = 28), started before ablation			Primary endpoint: change in LAEAT volume (no between-group difference); 1-year freedom from AF/AFl was secondary		1-year freedom from AF/AFl: 81% (95% CI 62–91) vs. 54% (34–70)		Log-rank *p* = 0.007 for freedom from AF/AFl; primary LAEAT endpoint neutral	Small trial
(**C**)										
**Study**	**Design and Population**		**Endpoint/Analysis**			**Main Finding**		**Relationship to AF**		**Principal Limitation**
Solomon et al., 2024 [44]	Prespecified echocardiographic analysis of STEP-HFpEF programme; 491 participants (253 semaglutide, 238 placebo)		Cardiac structure and function; AF burden not measured			At 52 weeks, less increase in LA volume: treatment difference −6.13 mL (95% CI −9.85 to −2.41); *p* = 0.0013		Supports a potentially favourable effect on the atrial substrate		Surrogate structural outcomes; no systematic rhythm assessment; cannot demonstrate AF prevention
Verma et al., 2024: STEP-HFpEF inflammation analysis [30]	Prespecified pooled analysis of two RCTs; 1145 participants		Change in CRP			Greater CRP reduction with semaglutide than placebo		Supports a potential anti-inflammatory mechanism		No evidence that CRP reduction mediates AF prevention
Manubolu et al., 2024 [42]	Semaglutide imaging substudy/STOP randomised-trial analysis in T2D		Epicardial adipose-tissue change			Reduction in epicardial adipose tissue reported with semaglutide		Imaging evidence concerning EAT; no demonstrated effect on atrial inflammation or AF		Small imaging analysis; no AF endpoint; cannot separate local from systemic weight-loss effects
Morano et al., 2015 [40]	Prospective study; 25 patients with T2D (12 exenatide, 13 liraglutide); 3 months		Visceral and epicardial fat assessed by ultrasound			Ultrasound fat thickness decreased at assessed sites (*p* < 0.001)		Indirect class-level mechanistic support		Not semaglutide-specific; small study; surrogate outcome
Dutour et al., 2016 [41]	Randomised trial; 44 patients with obesity and T2D; exenatide vs. reference treatment for 26 weeks		Epicardial and hepatic fat assessed by MRI/MRS			EAT decreased by 8.8% vs.. 1.2% (*p* = 0.003); hepatic fat also decreased; no significant myocardial triglyceride change		Supports biological plausibility for the GLP-1 RA class		Exenatide rather than semaglutide; no atrial rhythm outcome
Maretty et al., 2025 [26]	Proteomic analysis of STEP 1/STEP 2; 1728 participants with paired samples after quality control		Circulating proteins, including adiponectin, leptin and inflammatory/metabolic pathways			Broad treatment-related proteomic changes; a subset persisted after adjustment for weight and HbA1c changes		Supports a systemic endocrine/inflammatory mechanism		Exploratory biomarkers; no AF endpoint; not direct evidence of local atrial or EAT effects
Dozio et al., 2019 [39]	Human EAT expression study; samples from 17 patients with CAD undergoing CABG		GLP-1 receptor expression and metabolic gene associations			GLP-1 receptor expression associated with genes for fatty acid oxidation and white-to-brown differentiation		Biological rationale for targeting EAT		Non-interventional; not semaglutide-specific; no causal or rhythm outcome
Sivalingam et al., 2024 [47]	Double-blind RCT; 60 participants with T2D and albuminuria; background empagliflozin and renin–angiotensin system inhibition		Semaglutide up to 1.0 mg weekly vs. placebo for 26 weeks; plasma aldosterone and renin were secondary endpoints			Lower aldosterone with semaglutide vs. placebo (*p* = 0.035); no significant renin change		Human evidence of a possible aldosterone-related mechanism; AF outcomes not assessed		Aldosterone analysis: 33 participants; many values below the detection limit; no demonstration of AF prevention
(**D**)										
**Study/Reference**	**Design/Population**		**Comparison**			**Outcome/Ascertainment**		**Main Finding**		**Key Limitation**
Baser et al., 2024 [52]	Retrospective cohort; 5926 treated patients with obesity plus untreated comparators		Pooled semaglutide/tirzepatide exposure vs. non-use			Recorded AF and arrhythmias		AF: 3.83% vs. 5.17%; arrhythmias: 3.49% vs. 4.14%; adjusted HR 0.92 applies to composite CVD		Mixed GLP-1 RA and dual GIP/GLP-1 RA exposure; residual confounding; not semaglutide-specific
Hsiao et al., 2022 [67]	Taiwanese real-world cohort in T2D		SGLT2 inhibitors vs. GLP-1 RAs			New-onset AF		Lower AF risk with SGLT2 inhibitors		Non-randomised; no semaglutide-specific analysis
Thotamgari et al., 2022 [68]	FAERS pharmacovigilance analysis		GLP-1 RAs, DPP-4 inhibitors and SGLT2 inhibitors			AF adverse-event reporting		Drug-specific differences in spontaneous AF reporting		Reporting bias; incidence and causality cannot be assessed
Zhuo et al., 2022 [69]	Routine-care cohort of adults aged ≥66 years with T2D		SGLT2 inhibitors vs. DPP-4 inhibitors or GLP-1 RAs			Incident AF		Lower AF risk with SGLT2 inhibitors		Observational, class-level comparison

AF, atrial fibrillation; AFl, atrial flutter; CABG, coronary artery bypass grafting; CAD, coronary artery disease; CI, confidence interval; CRP, C-reactive protein; EAT, epicardial adipose tissue; ECG, electrocardiogram; FAERS, US Food and Drug Administration Adverse Event Reporting System; GIP, glucose-dependent insulinotropic polypeptide; GLP-1 RA, glucagon-like peptide-1 receptor agonist; HFpEF, heart failure with preserved ejection fraction; HR, hazard ratio; LA, left atrial; LAEAT, left atrial epicardial adipose tissue; NR, absolute events not reproduced in this summary; MRI, magnetic resonance imaging; MRS, magnetic resonance spectroscopy; OR, odds ratio; RCT, randomised controlled trial; RR, risk ratio; T2D, type 2 diabetes. The tables summarise studies directly informing atrial rhythm outcomes or relevant mechanisms. Overlapping trial populations are not independent evidence, and most atrial arrhythmia outcomes were secondary, post hoc, or adverse-event based. Observational and pharmacovigilance data do not establish causality or incidence.

**Table 3:** Registered rhythm-focused trials.

Trial	Design/Population	Intervention	Planned Endpoints and Rhythm Ascertainment	Registry Status (Latest Record Date)	Interpretation
DUTCH-WAIST, NCT06184633/EUCT 2022–501392–81–00 [72,73]	Multicentre, double-blind, randomised phase 4 trial in obesity and AF	Semaglutide 2.4 mg weekly vs. placebo; lifestyle intervention in both groups	At 12 months: 7-level rhythm-control hierarchy and binary composite of persistent AF, AF ablation, arrhythmic death or serious AAD-related events (Dutch record)	Recruitment stopped (Dutch registry, 25 August 2026); older CT.gov record still recruiting (19 September 2024)	No posted results; recruitment closure is not trial withdrawal
SEMINAL-AF, NCT06499857 [74]	Randomised phase 3 trial; obesity with paroxysmal or persistent AF	Semaglutide 2.4 mg vs. placebo, allocated 2:1	Primary: time to first AF event and AF-burden change through week 68; Holter, mobile telemetry or ILR	Recruiting (14 November 2025)	Prospective semaglutide-specific rhythm endpoints; no posted results
GOAL-AF, NCT05221229 [57,76]	Prospective, non-randomised feasibility study in obesity and first AF ablation; registry records 29 participants	Liraglutide plus risk-factor modification	Primary: adherence, recruitment and weight loss; AF outcomes and atrial imaging are additional assessments	Active, not recruiting (28 July 2025)	Published feasibility protocol; liraglutide, not semaglutide; no posted results
Semaglutide to Reduce AF Burden, NCT05209165 [80]	Randomised phase 4 trial in overweight or obesity with paroxysmal or early persistent AF	Semaglutide 2.4 mg vs. placebo	Primary: AF-burden change from baseline to weeks 50–52; ILR or 2-week ambulatory monitor if ILR declined	Suspended: company decision (27 October 2022)	No posted results; suspended does not mean completed or withdrawn
SOCRATES-AF, NCT04885634 [79]	Randomised phase 3 feasibility pilot; symptomatic paroxysmal or early persistent AF	Semaglutide 2.4 mg vs. placebo plus standard AF care	Primary: recruitment, follow-up completeness and resource needs; arrhythmia outcomes secondary, with ICM	Withdrawn: funding not received; actual enrolment 0 (3 November 2023)	Unrealised protocol; should not be described as ongoing evidence
WAIT, NCT07275697 [78]	Randomised phase 4 pre-ablation trial; assessor masked; planned enrolment 200	Semaglutide started ≥ 3 months before first AF ablation vs. standard care	Primary: 12-month freedom from AF/AFl/AT ≥ 30 s on ILR after a 3-month blanking period	Recruiting (10 August 2026)	No posted results; study is not withdrawn

AF, atrial fibrillation; AFl, atrial flutter; AT, atrial tachycardia; AAD, antiarrhythmic drug; ICM, implantable cardiac monitor; ILR, implantable loop recorder. Records were accessed on 14 September 2026; dates in the status column identify the latest registry update. Status reflects the available record, which may lag study conduct. DUTCH-WAIST uses the more recent Dutch CTIS-derived record alongside its older ClinicalTrials.gov record. Registered studies without published results are not included as completed efficacy evidence.

## Data Availability

No new data were created or analysed in this study. The original contributions presented in this study are included in the article. Further inquiries can be directed to the corresponding author(s).

## Abbreviations

AAD	antiarrhythmic drug;
AF	atrial fibrillation;
AFl	atrial flutter;
BMI	body mass index;
CAD	coronary artery disease;
CRT-D	cardiac resynchronisation therapy defibrillator;
CVD	cardiovascular disease;
EAT	epicardial adipose tissue;
GLP-1 RA	glucagon-like peptide-1 receptor agonist;
HF	heart failure;
HFpEF	heart failure with preserved ejection fraction;
HFrEF	heart failure with reduced ejection fraction;
ICD	implantable cardioverter-defibrillator;
LAEAT	left atrial epicardial adipose tissue;
MACE	major adverse cardiovascular events;
RCT	randomised controlled trial;
SGLT2	sodium–glucose cotransporter 2;
T2D	type 2 diabetes.
